# Supplementary material for: Dual heterogeneous structures lead to ultrahigh strength and uniform ductility in a Co-Cr-Ni medium-entropy alloy
Source: Nat Commun. 2020 May 13;11:2390. doi: 10.1038/s41467-020-16085-z (PMC7220923; doi:10.1038/s41467-020-16085-z)
Supplement: Supplementary file 1 — Supplementary Information [file 41467_2020_16085_MOESM1_ESM.pdf]

Supplementary information for

Dual heterogeneous structures lead to ultrahigh strength and uniform ductility in a Co-Cr-Ni medium-entropy alloy

Du et al.

Supplementary Note 1. Material Design

The deviation from the component of equi-atomic Ni-Co-Cr ternary alloy, namely, increasing Co content decreasing Ni content is to reduce the stacking fault energy (SFE) value of the FCC-structured matrix[1,2]. The Co-rich compositions are desirable owing to the following purposes (1) To intensify strain strengthening by forming high-density planar defects during the cold deformation process. (2) To suppress the recrystallization owing to the increased apparent activation energy of recrystallization[3]. (3) To promote the precipitation of γ' phase *via* decreasing the solubility of Al and Ti in the γ matrix[4]. In order to avoid the formation of brittle BCC phase only moderate contents of 3 at% Al and 3 at% Ti are added to precipitate the γ' phase during the aging process.

Supplementary Note 2. Estimation of the strengthening effect of the hierarchical precipitates

Due to the near fully coherent relationship of FCC/$L1_2$ and ultrafine size, the precipitation strengthening $L1_2$ phase is substantially sheared by the gliding dislocations during the deformation. Hence, the strengthening effects of $L1_2$ phase $\Delta\sigma_P$ are from three contributing factors, i.e., coherency strengthening $\Delta\sigma_C$, modulus mismatch strengthening $\Delta\sigma_M$ and ordering strengthening $\Delta\sigma_O$. The $\Delta\sigma_C$ and $\Delta\sigma_M$ play a part prior to the shearing, while the last one makes a contribution during the shearing. As a result, the $\Delta\sigma_P$ is equal to the larger one of ($\Delta\sigma_C$ and $\Delta\sigma_M$) and $\Delta\sigma_O$, of which can be expressed as[5]:

$$\Delta\sigma_C = M\cdot\alpha_\varepsilon(G\cdot\varepsilon)^{3/2}\left(\frac{rf}{0.5Gb}\right)^{1/2} \quad (1)$$

$$\Delta\sigma_M = M\cdot 0.0055(\Delta G)^{3/2}\left(\frac{2f}{G}\right)^{1/2}\left(\frac{r}{b}\right)^{\frac{3m}{2}-1} \quad (2)$$

$$\Delta\sigma_O = 0.81M\frac{\gamma_{APB}}{2b}\left(\frac{3\pi f}{8}\right)^{1/2} \quad (3)$$

where the Taylor factor M=3.06, α_ε=2.06 are constants for polycrystalline FCC structure and G=87 GPa is the shear modulus of the matrix (taken from the $(CoCrNi)_{94}Al_3Ti_3$ medium-entropy alloy[6]). ΔG=87-77=10 GPa is the difference of shear modulus between FCC matrix and $L1_2$ precipitate[7]. m=0.85[8]. γ_{APB} is the antiphase boundaries energy. As reported by literature about the Ni-based alloys, γ_{APB}≈0.3 J/m[2][9], $b=\frac{\sqrt{2}}{2}\alpha_{matrix}$=0.252 nm is the Burgers vector of the dislocation. $\varepsilon=2/3(\Delta\alpha/\alpha)$ is the misfit between matrix and precipitate, r is the mean radius of precipitates and f is the volume fraction of precipitates, where n is the number density of the precipitates.

In this study, the size of $(Ni,Co)_3(Al,Ti)$ particles precipitated during the annealing process are larger than that formed during the aging process. So the overall precipitation strengthening effect contributed by these heterogeneous $(Ni,Co)_3(Al,Ti)$ particles can be divided into two parts, namely,

$$\Delta\sigma_P = \Delta\sigma_p^{I} + \Delta\sigma_p^{II} \quad (4)$$

$\Delta\sigma_p^I$ is contributed by $(Ni,Co)_3(Al,Ti)$ particles formed during the annealing process. Where ε^{II}=0.0056%, r=38.04 nm, f=13.25% (calculated from the APT results by using the level rule[10]), Using the above expressions, the values for $\Delta\sigma_C$, $\Delta\sigma_M$ and $\Delta\sigma_O$ are calculated as 1.45 MPa, 116.73 MPa and 585.22 MPa, respectively. As $\Delta\sigma_C + \Delta\sigma_M$ is far lower than $\Delta\sigma_O$, the $\Delta\sigma_p^I$ is derived from ordering strengthening.

Similarly, $\Delta\sigma_p^{II}$ is contributed by $(Ni,Co)_3(Al,Ti)$ particles formed during the aging process. ε^{I}=0.011%, r=2.07 nm and f=10.95%. So the values for $\Delta\sigma_C$, $\Delta\sigma_M$ and $\Delta\sigma_O$ are calculated as 0.85 MPa, 47.68 MPa and 530.14 MPa, respectively. As $\Delta\sigma_C + \Delta\sigma_M$ is far lower than $\Delta\sigma_O$, the $\Delta\sigma_p^I$ is also contributed by ordering strengthening.

Based on the above calculation, the precipitation strengthening effect of heterogeneous $L1_2$ phase is estimated to be about 1115 MPa.

Supplementary Table 1. Compositions, structures of matrix, and tensile properties of the HEAs and MEAs studied previously.

Compositions	Matrix	σ_y (MPa)	σ_u (MPa)	ε_{ue} (%)	Ref.
HfNbTaTiZr	BCC	1202	1295	2	11
		1303	1334	1.9	
		1145	1262	9.7	
		1600	1900	3	12
		1520	1520	1.7	
CoCrFeNiMn	FCC	1400	1750	4	13
		930	1030	2	
FeNiCoCu	FCC	1149	1402	5	14
$(FeNiCoCu)_{86}Al_7Ti_7$	FCC	1477	1849	3.4	
$Cr_{15}Fe_{20}Co_{35}Ni_{20}Mo_{10}$	FCC	1311	1410	10	15
		1212	1360	14	
		1028	1249	17	
		879	1194	22	
		799	1127	27	
$(FeCoNiCr)_{94}Ti_2Al_4$	FCC	645	1094	30	16
		1005	1273	15	
TaHfZrTi	BCC	1300	1500	2.4	17
$Ni_{1.5}Co_{1.5}CrFeTi_{0.5}$	FCC	1308	1384	4.01	18
$Fe_{25}Co_{25}Ni_{25}Al_{10}Ti_{15}$	FCC	1860	2520	5.2	19
$Al_{0.5}Cr_{0.9}FeNi_{2.5}V_{0.2}$	FCC	1570	1763	10	20
		1810	1905	9	
$Fe_{49.5}Mn_{30}Co_{10}Cr_{10}C_{0.5}$	FCC	1300	1500	1.5	21
$(FeCoNi)_{86}$-Al_7Ti_7	FCC	1028	1446	46.3	4

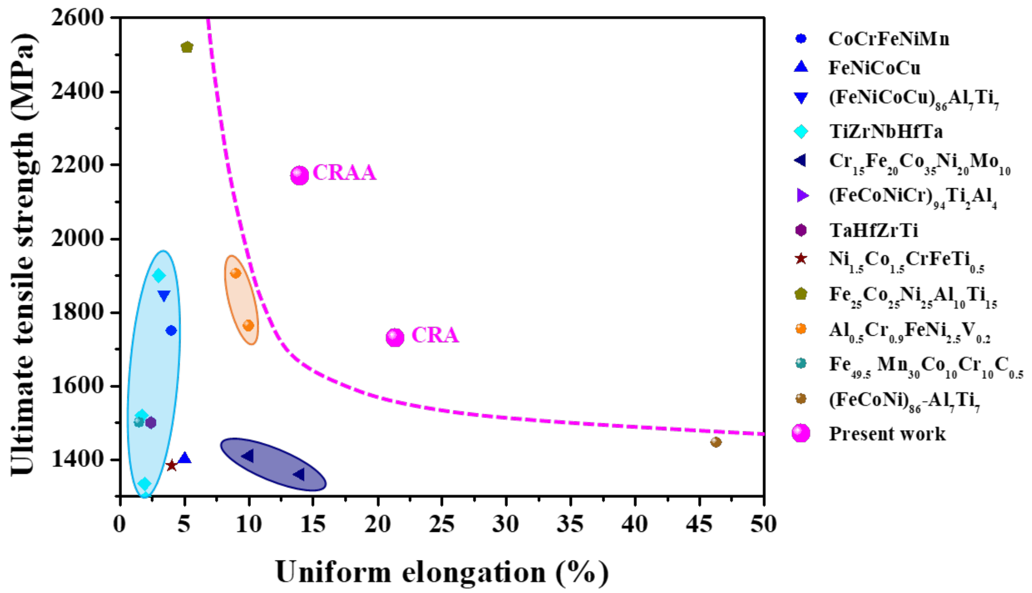

Supplementary Figure 1. Comparison of our developed alloys and other high strength MEAs and HEAs. Map of uniform elongation (ε_{ue}) versus ultimate tensile strength (UTS, σ_u) reveals the aged alloy achieves a combination of σ_u and ε_{ue} better than other high strength MEAs and HEAs using FCC or BCC as matrix. The data of the mechanical properties of these reported FCC-based materials are acquired from **Supplementary Table 1.**

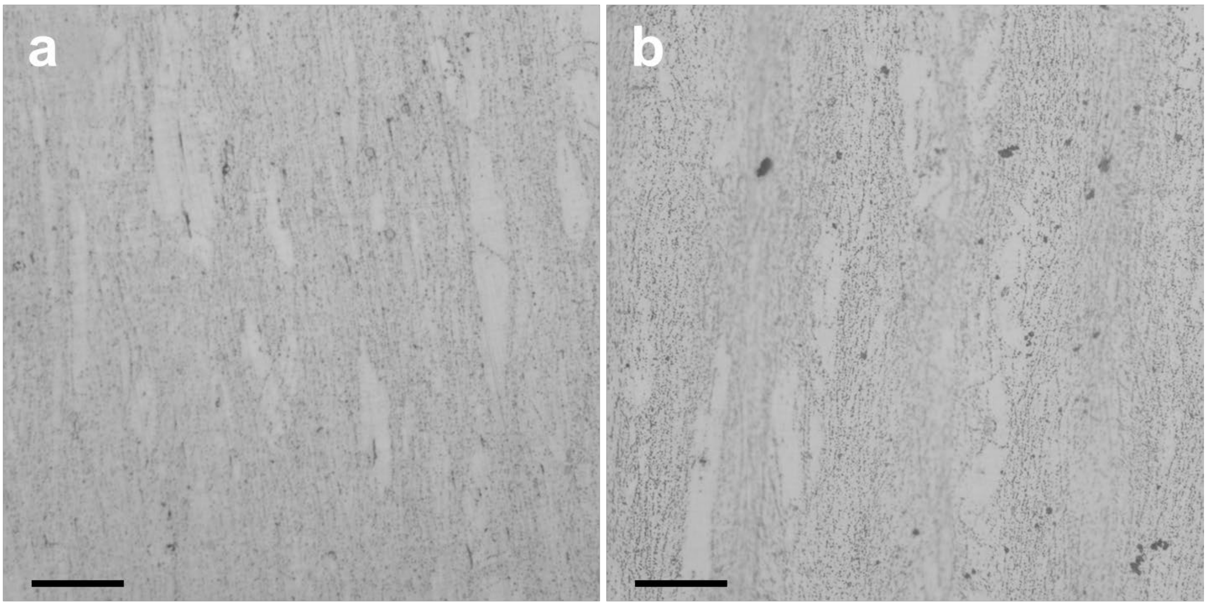

Supplementary Figure 2. Microstructural characterization for the (a) CRA and (b) CRAA alloys, revealing that many bands widely distributed in both CRA and CRAA alloys and surrounded by many fine grains. The scale bars in **(a)** and **(b)** are both 50 μm.

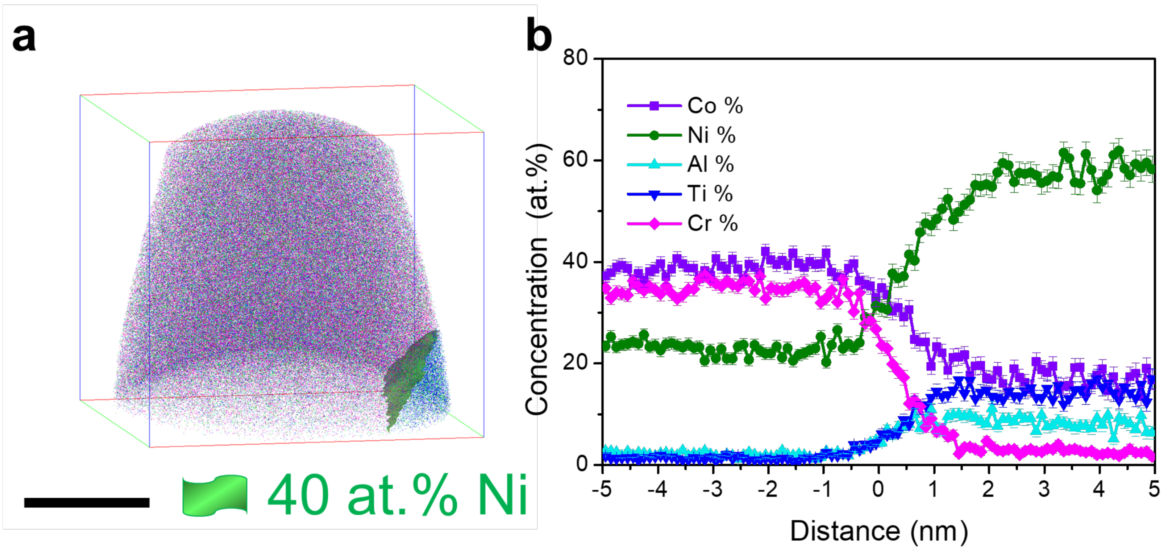

Supplementary Figure 3. APT results of the CRA alloy. **a**, the three-dimensional reconstruction of 40 at% Ni iso-concentration surfaces presenting the morphologies of a particle and matrix, respectively. Scale bar, 20 nm. **b**, one-dimensional concentration profile showing the element distributions from matrix to Ni-enriched particle. Error bars, s.d.

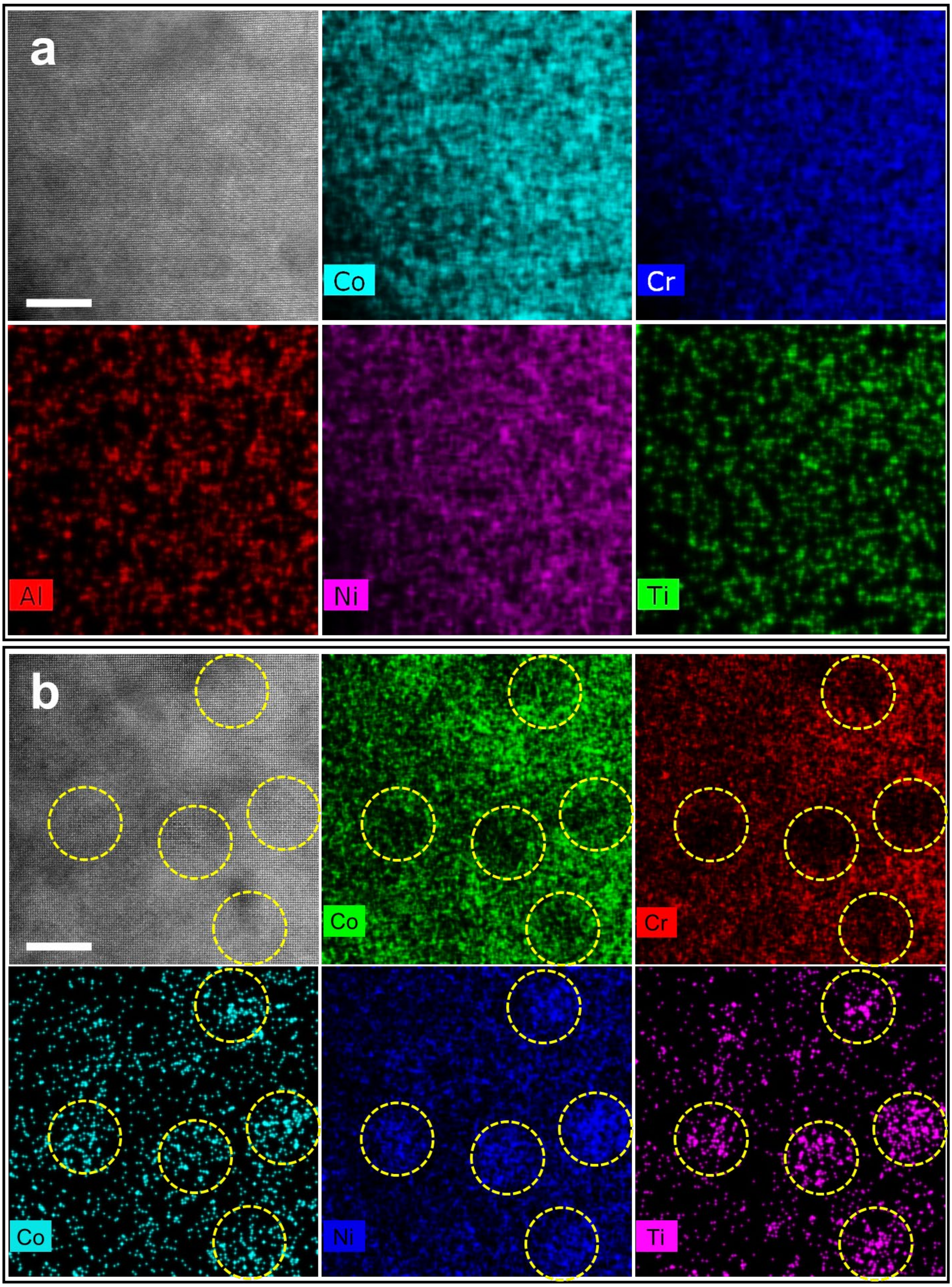

Supplementary Figure 4. Microstructure difference between CRA and CRAA alloys. Atomic-resolution HAADF-STEM image taken from [001] and corresponding EDS maps of **(a)** CRA and **(b)** CRAA alloys, showing that all five elements homogeneously distributed in CRA alloy while high-density $L1_2$ particles (with an average diameter of 4.14±0.62 nm) are precipitated in CRAA alloy. The scale bars in **(a)** and **(b)** are both 5 nm.

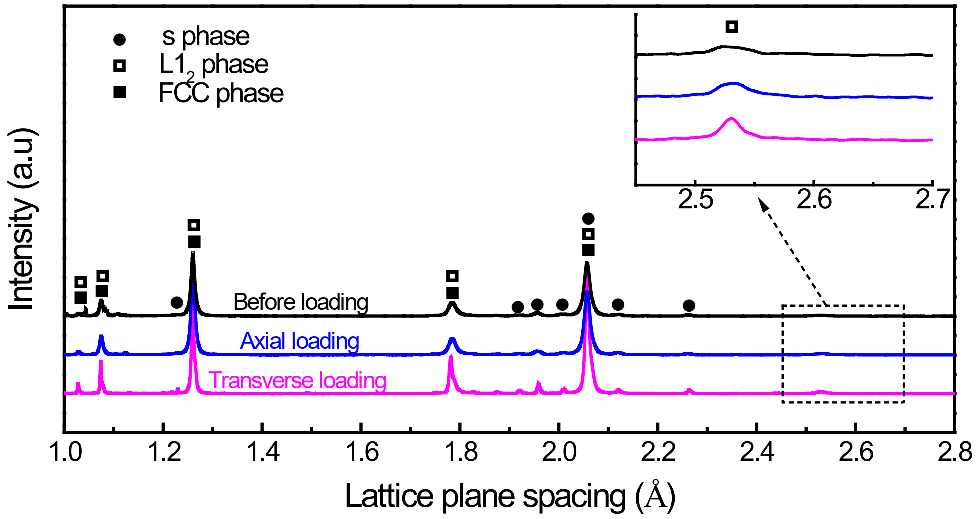

Supplementary Figure 5. Synchrotron X-ray diffraction (SXRD) spectrums of the without loaded, axially loaded and transversely loaded CRAA samples. It reveals that no phase transformation occurs during tensile process.

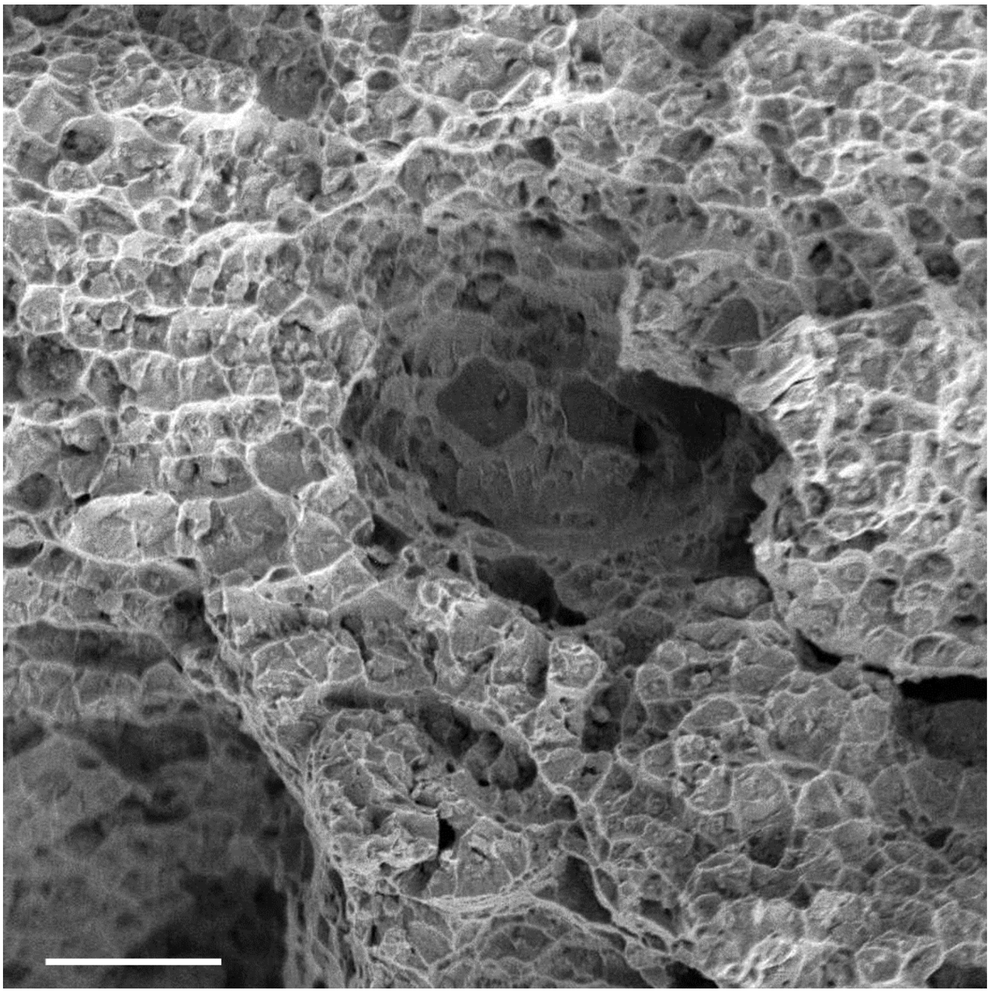

Supplementary Figure 6. The fracture morphology of the CRAA alloy. The SEM image shows obviously plastic deformation together with a micro-void coalescence fracture mode with fine dimples, indicating an excellent combination of ultra-high strength and good ductility. Scale bar, 5 μm.

Supplementary References

1 Heslop, J. Wrought nickel-chromium heat-resisting alloys containing cobalt. *Cobalt* **24**, 128-134, (1964).

2 Liu, S. F., Wu, Y., Wang, H. T., Lin, W. T., Shang, Y. Y., Liu, J. B., An, K., Liu, X. J., Wang, H. & Lu, Z. P. Transformation-reinforced high-entropy alloys with superior mechanical properties via tailoring stacking fault energy. *Journal of Alloys and Compounds* **792**, 444-455, (2019).

3 Yang, Z. a., Xiao, Y. & Shi, C. The role of cobalt in the high temperature creep of γ'-strengthened nickel-based superalloys. *Materials Science and Engineering A* **101**, 65-73, (1988).

4 Yang, T., Zhao, Y. L., Tong, Y., Jiao, Z. B., Wei, J., Cai, J. X., Han, X. D., Chen, D., Hu, A., Kai, J. J., Lu, K., Liu, Y. & Liu, C. T. Multicomponent intermetallic nanoparticles and superb mechanical behaviors of complex alloys. *Science* **362**, 933-937, (2018).

5 Ardell, A. J. Precipitation hardening. *Metallurgical Transactions A* **16**, 2131-2165, (1985).

6 Zhao, Y. L., Yang, T., Tong, Y., Wang, J., Luan, J. H., Jiao, Z. B., Chen, D., Yang, Y., Hu, A., Liu, C. T. & Kai, J. J. Heterogeneous precipitation behavior and stacking-fault-mediated deformation in a CoCrNi-based medium-entropy alloy. *Acta Materialia* **138**, 72-82, (2017).

7 Pollock, T. M. & Argon, A. S. Creep resistance of CMSX-3 nickel base superalloy single crystals. *Acta Metallurgica Et Materialia* **40**, 1-30.

8 Gerold, V. & Pham, H.-M. Precipitation hardening by misfitting particles and its comparison with experiments. *Scripta Metallurgica* **13**, 0-898.

9 Raynor, D. & Silcock, J. M. Strengthening Mechanisms in γ' Precipitating Alloys. *Metal Science Journal* **4**, 121-130, (1970).

10 Gardner, H., Pedrazzini, S., Douglas, J. O., De Lille, D., Moody, M. P. & Bagot, P. A. J. Atom Probe Tomography Investigations of Microstructural Evolution in an Aged Nickel Superalloy for Exhaust Applications. *Metallurgical and Materials Transactions A* **50**, 1862-1872, (2019).

11 Senkov, O. N. & Semiatin, S. L. Microstructure and properties of a refractory high-entropy alloy after cold working. *Journal of Alloys and Compounds* **649**, 1110-1123, (2015).

12 Schuh, B., Völker, B., Todt, J., Schell, N., Perrière, L., Li, J., Couzinié, J. P. & Hohenwarter, A. Thermodynamic instability of a nanocrystalline, single-phase TiZrNbHfTa alloy and its impact on the mechanical properties. *Acta Materialia* **142**, 201-212, (2018).

13 Shahmir, H., He, J., Lu, Z., Kawasaki, M. & Langdon, T. G. Effect of annealing on mechanical properties of a nanocrystalline CoCrFeNiMn high-entropy alloy processed by high-pressure torsion. *Materials Science and Engineering: A* **676**, 294-303, (2016).

14 Zheng, R., Chen, J., Xiao, W. & Ma, C. Microstructure and tensile properties of nanocrystalline (FeNiCoCu)1−xTixAlx high entropy alloys processed by high pressure torsion. *Intermetallics* **74**, 38-45, (2016).

15 Ming, K., Bi, X. & Wang, J. Precipitation strengthening of ductile Cr 15 Fe 20 Co 35 Ni 20 Mo 10 alloys. *Scripta Materialia* **137**, 88-93, (2017).

16 He, J. Y., Wang, H., Huang, H. L., Xu, X. D., Chen, M. W., Wu, Y., Liu, X. J., Nieh, T. G., An, K. & Lu, Z. P. A precipitation-hardened high-entropy alloy with outstanding tensile properties. *Acta Materialia* **102**, 187-196, (2016).

17 Huang, H., Wu, Y., He, J., Wang, H., Liu, X., An, K., Wu, W. & Lu, Z. Phase-Transformation Ductilization of Brittle High-Entropy Alloys via Metastability Engineering. *Adv Mater* **29**, (2017).

18 Moravcik, I., Cizek, J., Zapletal, J., Kovacova, Z., Vesely, J., Minarik, P., Kitzmantel, M., Neubauer, E. & Dlouhy, I. Microstructure and mechanical properties of Ni1,5Co1,5CrFeTi0,5 high entropy alloy fabricated by mechanical alloying and spark plasma sintering. *Materials & Design* **119**, 141-150, (2017).

19 Fu, Z. Q., Jiang, L., Wardini, J. L., MacDonald, B. E., Wen, H. M., Xiong, W., Zhang, D. L., Zhou, Y. Z., Rupert, T. J., Chen, W. P. & Lavernia, E. J. A high-entropy alloy with hierarchical nanoprecipitates and ultrahigh strength. *Sci. Adv.* **4**, 8, (2018).

20 Liang, Y. J., Wang, L., Wen, Y., Cheng, B., Wu, Q., Cao, T., Xiao, Q., Xue, Y., Sha, G., Wang, Y., Ren, Y., Li, X., Wang, L., Wang, F. & Cai, H. High-content ductile coherent nanoprecipitates achieve ultrastrong high-entropy alloys. *Nat Commun* **9**, 4063, (2018).

21 Su, J., Raabe, D. & Li, Z. Hierarchical microstructure design to tune the mechanical behavior of an interstitial TRIP-TWIP high-entropy alloy. *Acta Materialia* **163**, 40-54, (2019).
